# Factors Affecting School Performance in the Adolescents of USA- Youth Risk Behavior Surveillance System

**DOI:** 10.1192/j.eurpsy.2022.587

**Published:** 2022-09-01

**Authors:** M. Chahal, M. Telsem, B. Das, S. Patel, S. Gadiwala, R. Stuart, A. Mistry, T. Satnarine, P. Singla, A. Bakarr, P. Sharma, Y.-C. Hsieh, K. Aedma, S. Patel, R. Pathrose

**Affiliations:** 1 Government Medical Collelge, Department Of Psychiatry, Chandigarh, India; 2 Fulton State Hospital, Department Of Psychiatry, Fulton, United States of America; 3 Central New York Psychiatric center, Forensic Psychiatry, Marcy, United States of America; 4 Government Medical College,Surat, Department Of Paediatrics, Surat, India; 5 B J Medical College,Ahmedabad, Department Of Pediatrics, Ahmedabad, India; 6 Poznan University of Medical Science, Psychiatry, Poznań, Poland; 7 Dr N D Desai Medical College and Research Centre, Pediatrics, Nadiad, India; 8 Port of Spain General Hospital, Neonatology, Port of Spain, Trinidad and Tobago; 9 Government Medical College, Patiala, Paediatrics, Panchkula, India; 10 St George’s University school of Medicine, Medicine, True blue, Grenada; 11 Government Medical College, Amritsar, Department Of Psychiatry, Amritsar, India; 12 Icahn School of Medicine at Mount Sinai, School Of Public Health, New York City, United States of America; 13 UnityPoint Unitypoint Clinic Psychiatry, Department Of Psychiatry, Peoria, United States of America; 14 University of Illinois, Paediatrics, Chicago, United States of America; 15 Indiana University Memorial Health Ball Hospital, Department Of Psychiatry, Muncie, United States of America

**Keywords:** School Performance, Adolescents, mood and environment, diet and nutrition

## Abstract

**Introduction:**

Poor academic performance has been linked to factors such as sleep, health, illicit drug use, physical fighting, social media use, cyber bullying, physical activity, homelessness, times spent in video games and television. It is difficult to get a sense of the interplay between and relative importance of different behaviours/factors on academic performance as only limited research has been aimed at quantifying these factors.

**Objectives:**

To evaluate association of school performance and variables in five categories of the YRBSS: physical fighting, diet/lifestyle, electronic device usage, concurrent substance use, and violence/self-harm.

**Methods:**

The CDC Youth Risk Behavior Surveillance System (YRBSS) data from 1991-2019 was used in study. Respondents were grouped by good and poor school performance and variables related to nutrition/lifestyle, electronic device use, concurrent substance use, mood/violence/self-harm were analyzed using chi-square test.

**Results:**

A total of 41,235 student respondents.Nutrition/Lifestyle, electronic device use, concurrent substance use, mood/violence/self-harm are found to be significantly correlated with school performance.

**Table:** 

	Poor Performance n(%)	Good Performance n(%)	Total n(%)	p-Value
**Nutrition/Lifestyle**				
Daily breakfast	2,715(26)	11,429(38.22)	14,144(35.06)	<0.0001
Sodas ≥2/day	1,998(19.12)	2,710(9.03)	4,708(11.63)	<0.0001
**Concurrent Substance Use**				
Alcohol use	3,544(37.55)	8,067(28.49)	11,611(30.75)	<0.0001
Cigarette smoking	1,616(15.74)	1,845(6.17)	3,461(8.61)	<0.0001
**Mood/Violence/Self-Harm**				
Difficulty concentrating	4,188(46.34)	7,327(28.27)	11,516(32.94)	<0.0001
Felt sad or hopeless	4,373(41.06)	9,038(29.67)	13,410(32.62)	<0.0001
Considered suicide	2,567(24.14)	4,810(15.8)	7,377(17.96)	<0.0001

**Conclusions:**

In national data, we found school performance is affected by nutrition, lifestyle, substance use, mood and exposure to surrounding violence, and self-harm. Further studies should be planned to evaluate benefits from the risk stratification to reduce this burden amongst US adolescents.

**Disclosure:**

No significant relationships.

